# Supplementary material for: Resolving the full spectrum of human genome variation using Linked-Reads
Source: Genome Res. 2019 Apr;29(4):635–45. doi: 10.1101/gr.234443.118 (PMC6442396; doi:10.1101/gr.234443.118)
Supplement: Supplemental Material [file supp_gr.234443.118_Supplemental_longranger-2.2.zip › longranger-2.2.tar.bz2!/lr2.2-public/lib/stan/cmdstan/stan/doc/api/html/base__mcmc_8hpp_source.html]

Stan: src/stan/mcmc/base\_mcmc.hpp Source File


|  |  |
| --- | --- |
|  | Stan  2.5.0  probability, sampling & optimization |


- Main Page
- Related Pages
- Namespaces
- Classes
- Files

- File List
- File Members

All Classes Namespaces Files Functions Variables Typedefs Enumerations Enumerator Friends Macros Pages

- src
- stan
- mcmc

base\_mcmc.hpp

Go to the documentation of this file.

1 #ifndef STAN\_\_MCMC\_\_BASE\_MCMC\_\_HPP

2 #define STAN\_\_MCMC\_\_BASE\_MCMC\_\_HPP

3

4 #include <ostream>

5 #include <string>

6

7 #include <stan/mcmc/sample.hpp>

8

9 namespace stan {

10

11  namespace mcmc {

12

13  class base\_mcmc {

14

15  public:

16

17  base\_mcmc(std::ostream\* o, std::ostream\* e): out\_stream\_(o), err\_stream\_(e) {};

18

19  virtual ~base\_mcmc() {};

20

21  virtual sample transition(sample& init\_sample) = 0;

22

23  std::string name() { return name\_; }

24

25  virtual void write\_sampler\_param\_names(std::ostream& o) {};

26

27  virtual void write\_sampler\_params(std::ostream& o) {};

28

29  virtual void get\_sampler\_param\_names(std::vector<std::string>& names) {};

30

31  virtual void get\_sampler\_params(std::vector<double>& values) {};

32

33  virtual void write\_sampler\_state(std::ostream\* o) {};

34

35  virtual void get\_sampler\_diagnostic\_names(std::vector<std::string>& model\_names,

36  std::vector<std::string>& names) {};

37

38  virtual void get\_sampler\_diagnostics(std::vector<double>& values) {};

39

40  protected:

41

42  std::string name\_;

43

44  std::ostream\* out\_stream\_;

45  std::ostream\* err\_stream\_;

46

47  };

48

49  } // mcmc

50

51 } // stan

52

53 #endif

54

stan::mcmc::base\_mcmc::name\_

std::string name\_

**Definition:** base\_mcmc.hpp:38

stan::mcmc::base\_mcmc::get\_sampler\_diagnostic\_names

virtual void get\_sampler\_diagnostic\_names(std::vector< std::string > &model\_names, std::vector< std::string > &names)

**Definition:** base\_mcmc.hpp:35

stan::mcmc::base\_mcmc::get\_sampler\_params

virtual void get\_sampler\_params(std::vector< double > &values)

**Definition:** base\_mcmc.hpp:31

stan::mcmc::base\_mcmc::~base\_mcmc

virtual ~base\_mcmc()

**Definition:** base\_mcmc.hpp:19

stan::mcmc::base\_mcmc::out\_stream\_

std::ostream \* out\_stream\_

**Definition:** base\_mcmc.hpp:44

stan::mcmc::base\_mcmc::get\_sampler\_diagnostics

virtual void get\_sampler\_diagnostics(std::vector< double > &values)

**Definition:** base\_mcmc.hpp:38

stan::mcmc::base\_mcmc::write\_sampler\_params

virtual void write\_sampler\_params(std::ostream &o)

**Definition:** base\_mcmc.hpp:27

stan::mcmc::base\_mcmc::get\_sampler\_param\_names

virtual void get\_sampler\_param\_names(std::vector< std::string > &names)

**Definition:** base\_mcmc.hpp:29

stan::mcmc::base\_mcmc::base\_mcmc

base\_mcmc(std::ostream \*o, std::ostream \*e)

**Definition:** base\_mcmc.hpp:17

stan::mcmc::base\_mcmc::write\_sampler\_param\_names

virtual void write\_sampler\_param\_names(std::ostream &o)

**Definition:** base\_mcmc.hpp:25

stan::mcmc::base\_mcmc::name

std::string name()

**Definition:** base\_mcmc.hpp:23

stan::mcmc::base\_mcmc::err\_stream\_

std::ostream \* err\_stream\_

**Definition:** base\_mcmc.hpp:45

sample.hpp

stan::mcmc::base\_mcmc::transition

virtual sample transition(sample &init\_sample)=0

stan::math::e

double e()

Return the base of the natural logarithm.

**Definition:** constants.hpp:86

stan::mcmc::base\_mcmc::write\_sampler\_state

virtual void write\_sampler\_state(std::ostream \*o)

**Definition:** base\_mcmc.hpp:33

stan::mcmc::sample

**Definition:** sample.hpp:13

stan::mcmc::base\_mcmc

**Definition:** base\_mcmc.hpp:13


---

[ Stan Home Page ]

*© 2011–2014,
Stan Development Team.*
